# Predicting adverse outcomes in dilated cardiomyopathy using 3D echocardiography: penalised Cox regression versus machine learning

**DOI:** 10.1186/s12872-026-05645-8

**Published:** 2026-02-23

**Authors:** Manman Yang, Bingjie Qu, Jiacheng Cai, Danke Ma, Yan Zhang, Chengzeng Wang, Dahai Yu, Lin Liu

**Affiliations:** 1 Henan Institute of Interconnected Intelligent Health Management, Henan Key Laboratory of Chronic Disease Prevention and Therapy & Intelligent Health Management, The First Affiliated Hospital of Zhengzhou University, Zhengzhou, Henan 450052 China; 2 Department of Ultrasound, The First Affiliated Hospital of Zhengzhou University, Zhengzhou, Henan 450052 China; 3 School of Medicine, Keele University, Keele, Staffordshire, ST5 5BG United Kingdom

**Keywords:** Dilated cardiomyopathy, Risk stratification, Machine learning, Three-dimensional echocardiography, Survival prediction, Clinical decision support

## Abstract

**Background:**

Risk prediction in dilated cardiomyopathy (DCM) remains suboptimal, and there is uncertainty about how newer machine-learning (ML) methods compare with conventional regression for clinically useful prognostic modelling. Advanced three-dimensional (3D) echocardiographic measures, particularly of right ventricular function, may improve model performance when combined with routinely collected clinical data. We aimed to compare conventional Cox regression, penalised Cox regression, and ML approaches for prognostic modelling in DCM and to identify models that offer the best balance of discrimination, calibration, and interpretability for risk stratification.

**Methods:**

We conducted a retrospective cohort study including 196 adults with DCM attending a tertiary cardiology centre between 2021 and 2023. Participants were followed for a composite outcome of all-cause mortality, heart failure rehospitalisation, or left ventricular assist device (LVAD) implantation. We considered 41 candidate predictors, including demographic and clinical variables and 3D echocardiographic parameters (e.g. 4D right ventricular ejection fraction [4D-RVEF], tricuspid annular plane systolic excursion [TAPSE], right ventricular global longitudinal strain [RVGLS], left atrial volume index [LAVI], and pulmonary artery systolic pressure [PASP]). Twelve prognostic models were developed including conventional Cox regression, penalised Cox regression (Lasso-Cox), and several ML models—and evaluated using internal and performance assessment at different prediction horizons (up to 24 months). Performance was assessed using area under the receiver operating characteristic curve (AUC), calibration plots, and SHAP-based feature importance.

**Results:**

At 12 months, he best-performing ML model achieved the highest discrimination (AUC 0.990),followed by GBDT and Lasso-Cox (AUC 0.825). Model discrimination attenuated at longer prediction horizons, with the Lasso-Cox model maintaining acceptable performance at 24 months (AUC 0.729). Although RF and GBDT demonstrated excellent discrimination, calibration analyses revealed systematic under- and over-prediction at the extremes of risk. By contrast, Lasso-Cox showed more stable and favourable calibration across risk deciles. Across models, key predictors consistently included 4D-RVEF, LAVI, PASP, and TAPSE.

**Conclusions:**

In this DCM cohort, ML models, particularly RF, maximised discrimination but exhibited calibration issues. A penalised regression model (Lasso-Cox) provided the best overall trade-off between discrimination, calibration, and interpretability, and is therefore recommended as the preferred approach for clinical risk stratification and future public health–oriented implementation studies in DCM.

**Supplementary Information:**

The online version contains supplementary material available at 10.1186/s12872-026-05645-8.

## Background

Dilated cardiomyopathy (DCM), a leading cause of heart failure (HF), is defined by left-ventricular dilatation and systolic dysfunction that cannot be fully explained by abnormal loading conditions (e.g., hypertension or valvular disease) or by coronary heart disease [1–3]. It significantly contributes to HF prevalence, yet supporting epidemiologic data is sparse. Patients with DCM often experience recurrent hospitalizations and a high risk of mortality, with five-year survival rates approximating 50% [4, 5]. This substantial clinical burden underscores the necessity for effective prognostic tools to guide management and improve outcomes.

Traditional prognostic models in DCM have primarily relied on clinical assessments and two-dimensional echocardiographic parameters [6, 7]. Unlike conventional two-dimensional indices that reflect regional motion, three-dimensional RVEF directly quantifies global right ventricular pump function. This provides clinically relevant prognostic information and helps distinguish patients with similar LVEF but different risks of adverse outcomes. However, these models often lack the precision needed for individualized risk stratification. Advancements in three-dimensional (3D) echocardiography have enabled more accurate and comprehensive evaluation of cardiac structures and functions. Notably, 3D right ventricular ejection fraction (RVEF) has emerged as a robust predictor of adverse outcomes in DCM patients [8]. Despite these advancements, the integration of 3D echocardiographic measures into prognostic models remains limited.

The advent of machine learning (ML) techniques offers the potential to enhance prognostic modeling by capturing complex, nonlinear relationships among variables [9]. Studies have demonstrated that ML algorithms can outperform traditional statistical models in predicting outcomes in HF populations [10–12]. However, their performance relative to conventional statistical models in DCM, particularly when incorporating advanced echocardiographic parameters, remains unclear. Therefore, we compared traditional regression-based approaches with multiple ML algorithms within the same cohort to evaluate and contrast their prognostic performance.

In this study, we aimed to develop and compare traditional statistical models and ML algorithms for prognostic prediction in DCM patients, integrating 3D echocardiographic measures and routine clinical data. We evaluated model performance in terms of discrimination and calibration and examined performance at a longer prediction horizon (24 months). Our goal was to identify a prognostic model that balances accuracy and clinical applicability, thereby facilitating personalized risk stratification and management in DCM.

## Methods

A retrospective cohort incorporating total of 196 patients aged 18 to 80 years, diagnosed with Dilated Cardiomyopathy(DCM) according American College of Cardiology (ACC) guidelines [13], were included. The diagnosis of DCM was established based on the presence of left ventricular dilation and systolic dysfunction (LVEF < 50%) in the absence of abnormal loading conditions (such as hypertension or valvular disease) or significant coronary artery disease, as assessed by comprehensive clinical evaluation, echocardiography, and relevant laboratory and imaging investigations. All patients were recruited and treated at two campuses (the East Campus and the Hexi Campus) of Zhengzhou University First Affiliated Hospital between January. 2021 and December 2023. Demographic characteristics, anthropometric measurements, comorbidities, and prescription information were extracted from the hospital’s electronic health record (EHR) system and reviewed by a consultant cardiologist. Echocardiographic data were collected by experienced sonographers using standardized protocols. Quality control measures included periodic checks for data accuracy, identification and exclusion of obvious outliers, and re-measurement or correction of questionable values to ensure data quality and accuracy. Patients were followed up for at least 6 months, and the final follow-up date was January 1, 2025.

The research protocol was approved by the Clinical Research Ethics Committee of the First Affiliated Hospital of Zhengzhou University (ethics approval number: 2023-KY-1496), and all participants provided written informed consent before enrollment. Clinical trial number: not applicable.

The primary outcome was a composite clinical event occurring after hospital admission, which included left ventricular assist device (LVAD) implantation, rehospitalization due to heart failure, and all-cause mortality. Outcomes were confirmed and recorded by the attending physicians.

Candidate predictors were collected at enrollment and comprised demographic characteristics (age and sex), anthropometric measurements (including body mass index [BMI], systolic blood pressure [SBP], diastolic blood pressure [DBP], and heart rate [HR]), as well as clinical comorbidities such as New York Heart Association (NYHA) class > III, hypertension, and diabetes. Baseline medication usage was also recorded, including angiotensin-converting enzyme inhibitors or angiotensin receptor blockers (ACEI/ARB), β-blockers, mineralocorticoid receptor antagonists (MRA), sodium–glucose cotransporter-2 (SGLT2) inhibitors, loop diuretics, and digoxin. In addition, a comprehensive set of echocardiographic parameters was assessed by trained cardiologists following standardized protocols using the GE Vivid E95 color Doppler ultrasound diagnostic system (GE Healthcare, Vingmed Ultrasound, Horten, Norway). The system was equipped with a 4Vc-D cardiac probe (frequency 1.4–5.2 MHz) and the EchoPAC 204 workstation (GE Healthcare, Vingmed Ultrasound, Horten, Norway) for image storage and analysis. An electrocardiogram was recorded synchronously, with subjects in the left lateral or supine position. Patients were instructed to breathe calmly and hold their breath when necessary. Apical four-chamber cardiac view images were obtained for at least three cardiac cycles. All routine LV parameters were measured in accordance with current guidelines [14] .Left atrial (LA) volume was calculated using Simpson’s biplanar method from the apical four-chamber and two-chamber views and adjusted to the LA volume index (LAVI) based on body surface area. Right ventricular structures were also extensively evaluated, including basal and mid diameters (RVDd-base, RVDd-mid), and longitudinal diameter (RVLd). Right ventricular fractional area change (RVFAC) was determined by quantifying the RV end-diastolic and end-systolic areas. Tricuspid annular plane systolic excursion (TAPSE) was assessed using M-mode ultrasound. Tricuspid annular peak systolic velocity (TV-S′) was assessed using tissue Doppler imaging, and the right ventricular index of myocardial performance (RVIMP) was calculated. Pulmonary artery systolic pressure (PASP) was estimated using the gradient between the right ventricle and the right atrium (RA), derived from the tricuspid regurgitation jet, in conjunction with the RA pressure estimated from the diameter and collapsibility of the inferior vena cava.

The 2D strain software (EchoPAC-Advanced Functional Imaging RV analysis package) was employed to manually delineate the junctions of the tricuspid annulus with the RV free wall and the interventricular septum, as well as the apex of the RV endocardium in the apical RV-focused view. The width of the speckle-tracking region of interest was adjusted to encompass the entire myocardial wall while excluding the pericardium. The system automatically calculated the right ventricular global longitudinal strain (RVGLS) and the right ventricular longitudinal strain of the free wall (RVFWLS) [15, 16]. Four-dimensional (4D) echocardiography: In the apical RV-focused view, the gain, depth, and angle of the image were optimized, and the frame rate was set to exceed 40% of the subject’s heart rate. The software automatically identified and traced the RV endocardium, with manual adjustments made to the endocardial boundary until satisfactory tracking was achieved. The 4D functional and volume parameters, as well as the time-volume change curve of the right ventricle, were obtained. Parameters including 4D-RVEF, right ventricular stroke volume (RVSV), end-diastolic volume (RVEDV), end-systolic volume (RVESV), 4D-TAPSE, and 4D-RVFAC were measured [17]. RVSV, RVEDV, and RVESV were normalized for body surface area to derive the right ventricular stroke volume index (RVSVI), end-diastolic volume index (RVEDVI), and end-systolic volume index (RVESVI).

All variables were measured and documented at the time of patient admission. To ensure comparability across different scales and to facilitate interpretation of model coefficients, continuous predictors were standardized using Z-scores. Importantly, there were no missing values among the candidate predictors included in this analysis.

This study aimed to model clinical events within the first year using baseline predictors. Five modeling approaches were used to develop predictive models, including Cox regression, Lasso-Cox, Random Forest, Gradient Boosted Decision Tree (GBDT) and Support Vector Machine (SVM). To assess the independent association of each baseline predictor with clinical outcomes while adjusting for other baseline variables [18], a Record-wide association study (ReWAS) [19] was performed. Each predictor was entered individually into an adjusted regression model, and the resulting p-values were corrected for multiple testing using the Benjamini–Hochberg false discovery rate (FDR) procedure (5%). Predictors with adjusted *p*-values < 0.05 were considered to have stronger evidence of association and were used as candidate variables for subsequent model development. ReWAS was performed as a global pre-modelling screening step and was not included within resampling procedures.

Considering potential nonlinear relationships between continuous predictors and outcomes, the Fractional Polynomial (FP) transformation [20] method was applied. The optimal FP terms were selected based on the minimum Akaike Information Criterion (AIC) in the Cox models. The term “Non-linear transformer” refers to fractional polynomial (FP) transformation applied to continuous predictors prior to model fitting.

Given the moderate number of predictors relative to the sample size, a structured modeling framework was used to reduce overfitting and to evaluate alternative modeling assumptions rather than relying on a single modeling approach. Specifically, the analysis was designed to address two methodological dimensions: (1) functional form (linear versus nonlinear relationships between predictors and outcomes) and (2) modeling paradigm (conventional regression versus machine-learning approaches). Cox regression served as a baseline clinical prognostic model, and fractional polynomial transformation was used to explore potential nonlinear functional forms. Lasso-Cox represented penalized regression under linear assumptions. Machine-learning algorithms (random forest, gradient boosting decision tree, and support vector machine) were applied to capture nonlinear relationships and interactions among predictors. ReWAS-type analysis was used as a variable-screening procedure rather than as an independent predictive model. The Lasso-Cox model used 10-fold cross-validation to determine the optimal lambda (λ) value. In RF models, variable importance was used to quantify and rank predictor contributions. In GBDT and SVM models, SHAP (SHapley Additive exPlanations) [21, 22] values were calculated to interpret and rank the contributions of different predictors.

Several internal validation methods were employed to minimize potential overfitting, including bootstrapping (Cox models), cross-validation (Lasso and SVM), out-of-bag estimation (RF), and boosting (GBDT). A longer-horizon performance assessment was conducted in patients who were event-free during the first year and were subsequently followed into the second year of follow-up. Hyperparameters were determined within the internal resampling procedures of each modelling framework, and no post-hoc tuning based on validation performance was performed. Detailed hyperparameters and model configurations for all machine-learning models are provided in the Supplementary Tables.

Model discrimination was evaluated using the area under the receiver operating characteristic curve (AUC) [23] with 95% confidence intervals (CI). The optimal threshold for classification was determined based on the maximum sum of sensitivity and specificity. Model performance was further assessed by calculating sensitivity, specificity, and overall accuracy at the optimal threshold. Calibration curves were plotted to compare predicted probabilities with observed risks. Predicted probabilities were grouped into deciles and visually assessed using LOWESS smoothing [24].

All statistical analyses and model constructions were performed using R software (version 4.3.1). Key R packages used included survival [25], survminer [26], glmnet [27], randomForestSRC [28], and caret [29]. The statistical significance level was uniformly set at a two-sided α = 0.05. This study was reported in accordance with the TRIPOD-AI reporting guideline for prediction model development and validation.

## Results

A total of 196 patients with dilated cardiomyopathy were enrolled. Of these, 78 (39.8%) did not experience a composite endpoint event during follow-up, while 118 (60.2%) experienced at least one endpoint event (Table 1). The overall mean age was 51.06 ± 13.37 years, with the event and non-event groups having mean ages of 50.97 ± 12.78 and 51.19 ± 14.30 years, respectively, showing no significant difference (*P* = 0.911). The cohort was predominantly male (81.1%), with similar proportions in the event group (82.2%) and non-event group (79.5%; *P* = 0.772). Body mass index did not differ significantly between groups (24.69 ± 4.76 vs. 25.55 ± 4.48 kg/m²; *P* = 0.205). Systolic blood pressure, diastolic blood pressure, and heart rate were also comparable between groups.

**Table 1:** Descriptive statistics of the study population

	Total (*N* = 196)	Status		*P*-value
		Not event (*N* = 78)	Event (*N* = 118)	
Age, year (SD)	51.06 (13.37)	51.19 (14.30)	50.97 (12.78)	0.911
Male, (%)	159 (81.1)	62 (79.5)	97 (82.2)	0.772
Body Mass Index, kg/m² (SD)	25.03 (4.66)	25.55 (4.48)	24.69 (4.76)	0.205
Systolic Blood Pressure, mm Hg (SD)	116.62 (17.43)	119.47 (15.48)	114.74 (18.42)	0.062
Diastolic Blood Pressure, mm Hg (SD)	77.64 (13.44)	78.06 (13.73)	77.36 (13.29)	0.722
Heart Rate, beats/min (SD)	79.67 (14.14)	79.06 (13.58)	80.07 (14.54)	0.628
NYHA Class > III, (%)	150 (76.5)	52 (66.7)	98 (83.1)	0.013
Hypertension, (%)	64 (32.7)	31 (39.7)	33 (28.0)	0.117
Diabetes, (%)	46 (23.5)	12 (15.4)	34 (28.8)	0.046
ACEI/ARB Usage, (%)	163 (83.2)	63 (80.8)	100 (84.7)	0.594
Beta-blockers Usage, (%)	158 (80.6)	59 (75.6)	99 (83.9)	0.212
MRA Usage, (%)	146 (74.5)	57 (73.1)	89 (75.4)	0.840
SGLT2 Inhibitors Usage, (%)	152 (77.6)	58 (74.4)	94 (79.7)	0.486
Loop Diuretics Usage, (%)	173 (88.3)	68 (87.2)	105 (89.0)	0.875
Digoxin Usage, (%)	82 (41.8)	26 (33.3)	56 (47.5)	0.070
LVDd, mm (SD)	67.37 (9.64)	66.58 (9.63)	67.89 (9.64)	0.352
LVDs, mm (SD)	56.21 (9.82)	55.38 (10.01)	56.76 (9.70)	0.338
LVEDVI, ml/m² (SD)	130.49 (39.01)	125.68 (40.01)	133.67 (38.18)	0.161
LVESVI, ml/m² (SD)	94.45 (34.77)	89.53 (34.14)	97.70 (34.94)	0.108
LVEF, % (SD)	34.57 (13.54)	33.95 (13.06)	34.98 (13.89)	0.602
E/e′ (SD)	15.43 (7.45)	14.14 (7.54)	16.28 (7.31)	0.049
LAVI, ml/m² (SD)	50.70 (23.74)	44.41 (17.52)	54.85 (26.34)	0.002
MR ≥ Moderate, (%)	132 (67.3)	51 (65.4)	81 (68.6)	0.748
RVDd-base, mm (SD)	43.08 (8.16)	41.63 (6.66)	44.03 (8.92)	0.043
RVDd-mid, mm (SD)	32.30 (7.55)	32.33 (6.98)	32.27 (7.93)	0.955
RVLd, mm (SD)	80.49 (12.59)	78.44 (13.91)	81.86 (11.50)	0.063
TAPSE, mm (SD)	16.18 (3.62)	17.36 (3.58)	15.41 (3.44)	< 0.001
RVFAC, % (SD)	33.94 (9.80)	35.88 (10.65)	32.66 (9.01)	0.024
RVIMP (SD)	0.50 (0.10)	0.48 (0.10)	0.50 (0.11)	0.15
TV-S’, m/s (SD)	0.11 (0.06)	0.11 (0.03)	0.11 (0.08)	0.913
RVGLS, % (SD)	12.42 (4.21)	13.67 (3.97)	11.60 (4.18)	0.001
RVFWLS, % (SD)	15.59 (5.38)	17.33 (5.87)	14.44 (4.72)	< 0.001
TR ≥ Moderate, (%)	79 (40.3)	26 (33.3)	53 (44.9)	0.142
PASP, mm Hg (SD)	37.57 (13.96)	32.91 (13.41)	40.65 (13.50)	< 0.001
4D-RVEF, % (SD)	41.05 (8.22)	45.56 (6.44)	38.06 (7.93)	< 0.001
RVEDVI, ml/m² (SD)	102.51 (45.93)	101.42 (43.89)	103.24 (47.40)	0.787
RVESVI, ml/m² (SD)	48.90 (19.90)	45.76 (17.04)	50.97 (21.40)	0.073
1-RVESVI (SD)	-48.79 (19.92)	-45.76 (17.04)	-50.80 (21.45)	0.083
RVSVI, ml/m² (SD)	29.43 (9.38)	28.44 (8.39)	30.08 (9.97)	0.233
4D-TAPSE, mm (SD)	15.22 (4.03)	16.09 (3.80)	14.65 (4.09)	0.014
4D-RVFAC, % (SD)	32.07 (8.50)	34.33 (9.42)	30.57 (7.51)	0.002

*BMI* body mass index, *SBP* systolic blood pressure, *DBP* diastolic blood pressure, *HR* heart rate, *NYHA* New York Heart Association functional class, *ACEI* angiotensin-converting enzyme inhibitor, *ARB* angiotensin receptor blocker, *MRA* mineralocorticoid receptor antagonist, *SGLT2* sodium–glucose cotransporter-2 inhibitor, *LVDd* left ventricular end-diastolic diameter, *LVDs* left ventricular end-systolic diameter, *LVEDVI* left ventricular end-diastolic volume index, *LVESVI* left ventricular end-systolic volume index, *LVEF* left ventricular ejection fraction, *E/e*′ ratio of early mitral inflow velocity to mitral annular early diastolic velocity, *LAVI* left atrial volume index, *MR* mitral regurgitation, *RVDd-base* right ventricular basal diameter, *RVDd-mid* mid right ventricular diameter, *RVLd* right ventricular longitudinal diameter, *TAPSE* tricuspid annular plane systolic excursion, *RVFAC* right ventricular fractional area change, *RVIMP* right ventricular myocardial performance index, *TV-S*′ tricuspid annular systolic velocity, *RVGLS* right ventricular global longitudinal strain, *RVFWLS* right ventricular free-wall longitudinal strain, *TR* tricuspid regurgitation, *PASP* pulmonary artery systolic pressure, *4D-RVEF* four-dimensional right ventricular ejection fraction, *RVEDVI* right ventricular end-diastolic volume index, *RVESVI* right ventricular end-systolic volume index, *1-RVESVI* reciprocal right ventricular end-systolic volume index, *RVSVI* right ventricular stroke volume index, *4D-TAPSE* four-dimensional tricuspid annular plane systolic excursion, *4D-RVFAC*, four-dimensional right ventricular fractional area change

Baseline characteristics were summarized descriptively. *P*-values are provided for reference only and were not used for variable selection or causal interpretation

**Fig. 1:**
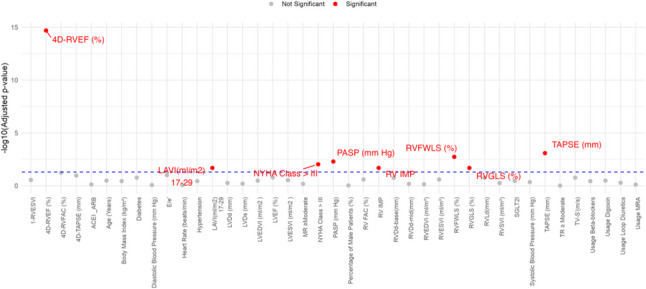
Manhattan plot adjusted for multiple comparisons using the Benjamini-Hochberg method. The y-axis shows the −log10 (adjusted *p*-value) after Benjamini–Hochberg false discovery rate correction. The horizontal dashed line indicates the significance threshold (FDR 5%). Predictors above this line are significantly associated with adverse outcomes

In the univariate ReWAS analysis, 8 of 41 candidate predictors reached significance after Benjamini - Hochberg correction. The transformation of $\:{\mathrm{log}}_{10}(\mathrm{a}\mathrm{d}\mathrm{j}\mathrm{u}\mathrm{s}\mathrm{t}\mathrm{e}\mathrm{d}\:P-\mathrm{v}\mathrm{a}\mathrm{l}\mathrm{u}\mathrm{e})$ for each variable is plotted in a Manhattan diagram, highlighting that 4D-RVEF, LAVI, PASP, TAPSE, RVFWLS, and RVGLS exceeded the threshold (Fig. 1).

Supplemental Table 1 presents the optimal FP transformations for continuous predictors. It lists the optimal FP terms for each variable selected by AIC and BIC values.

**Fig. 2:**
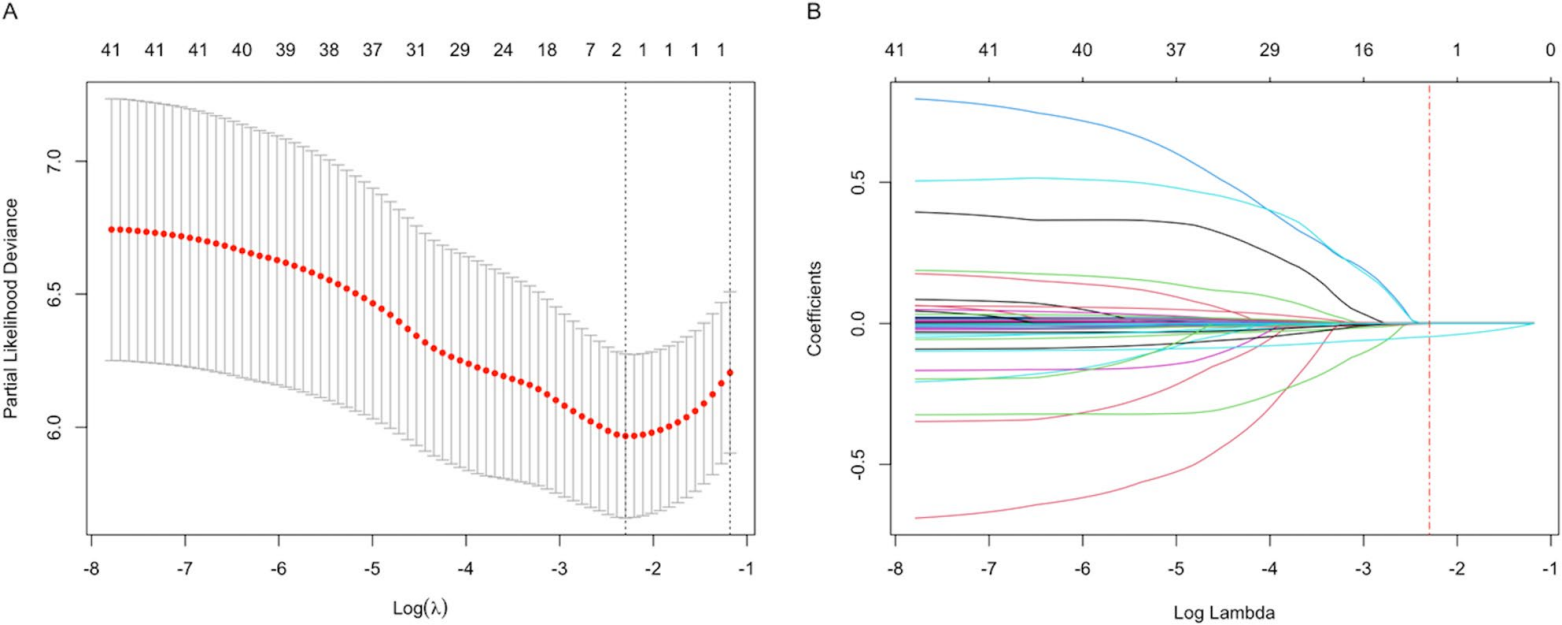
LASSO regularization path and cross-validation plot. **A** Ten-fold cross-validation curve showing partial likelihood deviance across penalty parameters (λ). The vertical dashed line indicates the optimal λ value selected by cross-validation, which balances model fit and overfitting. **B** Coefficient shrinkage paths for all candidate predictors. As the penalty increases, regression coefficients shrink toward zero and non-informative variables are removed from the model. The numbers above the plot represent the number of predictors retained at each λ. This procedure identifies a parsimonious set of clinically relevant predictors for risk estimation

The Lasso-Cox regularization path and cross-validation deviance curve (Fig. 2) presents model tuning. Partial likelihood deviance reached its minimum at log(λ). Coefficient shrinkage along the path identified the optimal penalization, balancing model complexity and fit.

For 12-month survival prediction, 12 models were evaluated (Table 2). The backward stepwise Cox regression (Supplemental Table 2) achieved a time-dependent AUC of 0.862 (95%CI: 0.789–0.935). The Lasso‐Cox model optimized via 10-fold cross-validation (lambda details in Supplemental Table 3) yielded an AUC of 0.825 (95%CI: 0.739–0.911). RF models built on all predictors and on ReWAS selected predictors obtained AUCs of 0.990 (95%CI: 0.980–1.000) and 0.962 (95%CI: 0.938–0.986), respectively. Incorporating fractional polynomial transformations, the non-linear transformer Cox model showed reduced discrimination (AUC 0.806; 95%CI: 0.719–0.893), whereas the non-linear transformer RF achieved an AUC of 0.988. GBDT models, with standard settings, a selected-variable variant trained on the top 10 most important predictors (GBDT selected), and ReWAS settings, demonstrated AUCs of 0.923 (95%CI: 0.886–0.961), 0.903 (95%CI: 0.859–0.947), and 0.828 (95%CI: 0.768–0.888), respectively. Because all models were evaluated within the same cohort, AUC values were interpreted as descriptive estimates of discrimination rather than formal statistical comparisons between algorithms.

**Table 2:** Overall predictive model performance metrics

Model	Time (Month)	AUC (95% CI)	Sensitivity	Specificity	Sensitivity + Specificity	Accuracy	Threshold
Cox	12	0.862 (78.91–93.53)	0.703	0.881	1.583	0.847	0.439
	24	0.745 (66.29–82.63)	0.337	0.965	1.302	0.699	1.151
Lasso-Cox	12	0.825 (73.85–91.10)	0.703	0.874	1.577	0.842	–2.834
	24	0.729 (64.54–81.23)	0.434	0.876	1.310	0.689	–2.861
Random Forest	12	0.990 (97.99–100.0)	0.946	0.981	1.927	0.974	0.303
	24	0.753 (67.44–83.21)	0.494	0.973	1.467	0.770	0.266
ReWAS + Random Forest	12	0.962 (93.83–98.57)	0.973	0.830	1.803	0.857	0.263
	24	0.795 (72.11–86.86)	1.000	0.673	1.673	0.811	0.10
Nonlinear-Cox	12	0.806 (71.94–89.35)	0.156	0.649	0.918	1.567	0.867
	24	0.685 (59.50-77.44)	-1.021	0.663	0.584	1.247	0.617
Non-linear transformer Random Forest	12	0.988 (97.62–100.0)	0.289	0.973	0.956	1.929	0.959
	24	0.723 (64.02–80.48)	0.281	0.482	0.965	1.447	0.760
ReWAS + Non-linear transformer Random Forest	12	0.965 (94.30-98.71)	0.277	0.946	0.874	1.820	0.888
	24	0.794 (72.00-86.71)	0.164	0.904	0.761	1.665	0.821
GBDT	12	0.923 (0.886–0.961)	0.946	0.899	1.845	0.908	0.120
	24	0.753 (0.673–0.833)	0.723	0.619	1.342	0.663	0.042
GBDT (Selected)	12	0.903 (0.859–0.947)	0.946	0.899	1.845	0.908	0.120
	24	0.722 (0.639–0.806)	0.723	0.619	1.342	0.663	0.042
ReWAS + GBDT	12	0.828 (0.768–0.888)	0.892	0.648	1.54	0.694	0.70
	24	0.725 (0.639–0.811)	0.699	0.531	1.23	0.602	0.641
SVM	12	0.676 (0.581–0.772)	0.7297	0.5484	1.2781	0.5833	–22.1186
	24	0.672 (0.583–0.760)	0.5783	0.6935	1.2719	0.6276	–22.0941
ReWAS + SVM	12	0.678 (0.585–0.772)	0.7297	0.5484	1.2781	0.5833	–22.1186
	24	0.681 (0.593–0.770)	0.5783	0.6935	1.2719	0.6276	–22.0941

Non-linear transformer refers to applying polynomial power transformations to numeric variables, *AUC* stands for Area Under the Curve

SVM models yielded the lowest AUCs (0.676 and 0.678), reflecting limited discriminative ability. Overall, RF based methods demonstrated the strongest predictive performance, followed by GBDT, whereas traditional Cox, Lasso-Cox and SVM models exhibited more modest accuracy.

To characterise model complexity and mitigate potential overfitting, key regularisation and structural constraints were prespecified for all machine-learning models. For the random survival forest (RSF) models (Supplemental Table 4), forests were grown using sampling without replacement (resample size = 124) with a minimum terminal node size of 15 and log-rank splitting. The number of candidate variables considered at each split (mtry) ranged from 3 to 8 depending on the feature set, and forests consisted of 500 trees (1000 trees for the non-linear transformed model). The average number of terminal nodes per tree was approximately 7–8, providing an estimate of the effective model complexity. Out-of-bag (OOB) prediction was used as an internal validation procedure and as a constraint against overfitting. Gradient boosted decision tree (GBDT) survival models were implemented using a Cox proportional hazards objective function (Supplemental Table 6). Model complexity was controlled by limiting the maximum tree depth to 6, applying shrinkage via a learning rate (eta) of 0.05, subsampling 70% of observations and 80% of predictors for each tree, and restricting the number of boosting rounds (500–800 depending on feature set). These hyperparameters act as regularisation to reduce variance and prevent overfitting. For the survival support vector machine (SVM), a regression-type survival SVM with an additive kernel was used, optimised via quadratic programming (Supplemental Table 7). Model flexibility was controlled by the regularisation parameter (gamma.mu = 0.1), and the effective complexity of the model is reflected by the number of retained support vectors.

**Fig. 3:**
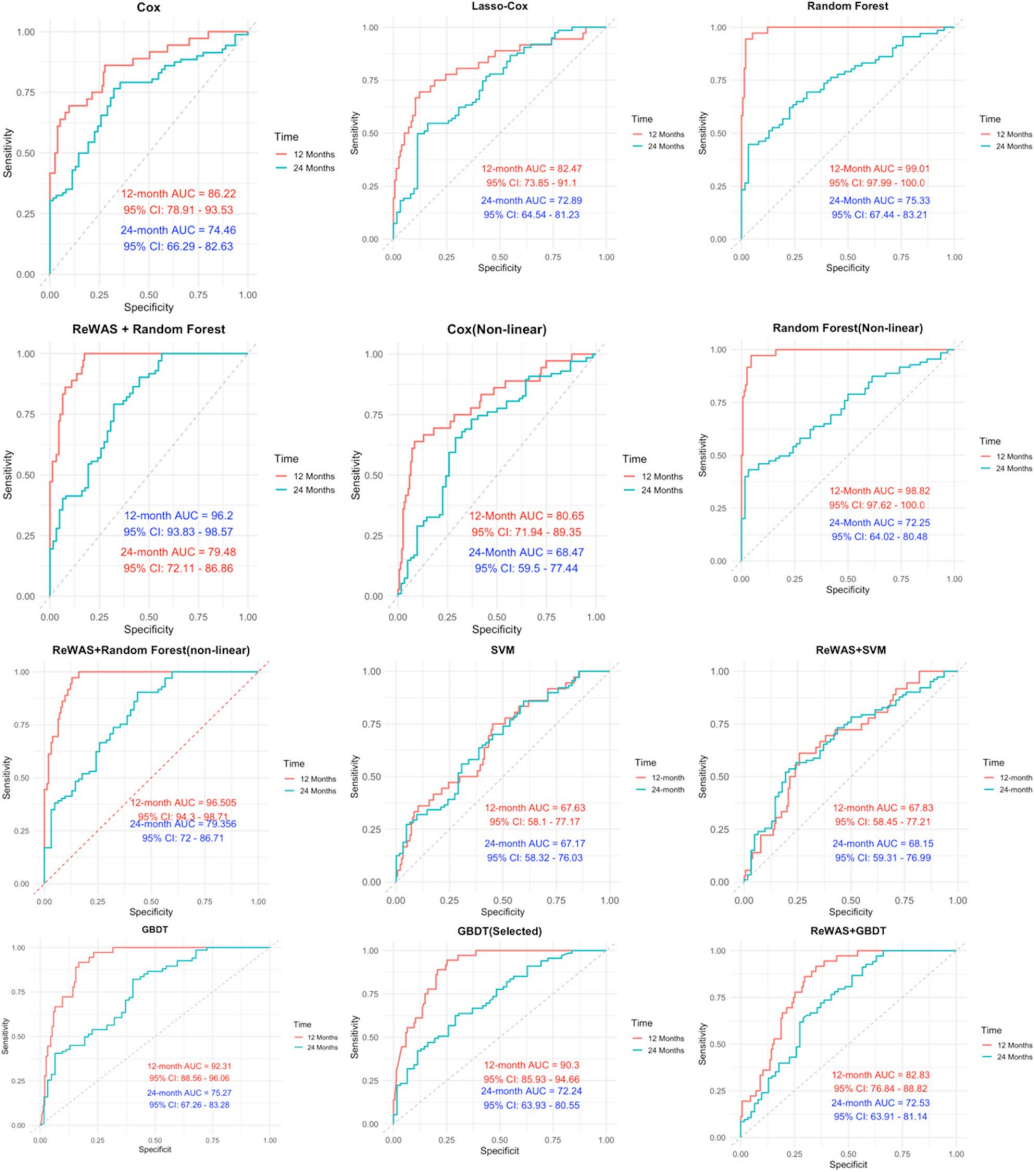
Comparison of ROC Curves for different Prediction Models at 12 and 24 Months.SVM stands for Support Vector Machine. GBDT stands for Gradient Boosted Decision Tree. ReWAS (Record-wide Association Study): used here to denote univariable screening with FDR correction for candidate predictor selection before model fitting

Performance at the longer follow-up horizon showed decreased discrimination across all models (Table 2; Fig. 3). The Cox regression model yielded a 24-month AUC of 0.745 (95%CI: 0.663–0.826), and the Lasso-Cox model achieved 0.729 (95%CI: 0.645–0.812). The standard RF model maintained high performance with an AUC of 0.753 (95%CI: 0.674–0.832), while its ReWAS selected variant improved to 0.795 (95%CI: 0.721–0.869). The non-linear transformer Cox model exhibited lower discrimination at 0.685 (95%CI: 0.595–0.774), whereas the non-linear transformer RF achieved 0.723 (95%CI: 0.640–0.805) and its ReWAS optimized counterpart reached 0.794 (95%CI: 0.720–0.867). Among gradient boosting methods, GBDT and GBDT-selected models recorded AUCs of 0.753 (95%CI: 0.673–0.833) and 0.722 (95%CI: 0.639–0.806), respectively, while the ReWAS + GBDT variant achieved 0.725 (95%CI: 0.639–0.811). SVM models showed the lowest 24-month AUCs, at 0.672 (95%CI: 0.583–0.760) for SVM and 0.681 (95%CI: 0.593–0.770) for ReWAS + SVM. Across all models, discrimination decreased at 24 months compared with the 12-month analysis. This suggests that baseline predictors were more informative for short-term prognosis, whereas longer-term outcomes may be increasingly influenced by disease progression and treatment modifications during follow-up rather than baseline characteristics alone.

**Fig. 4:**
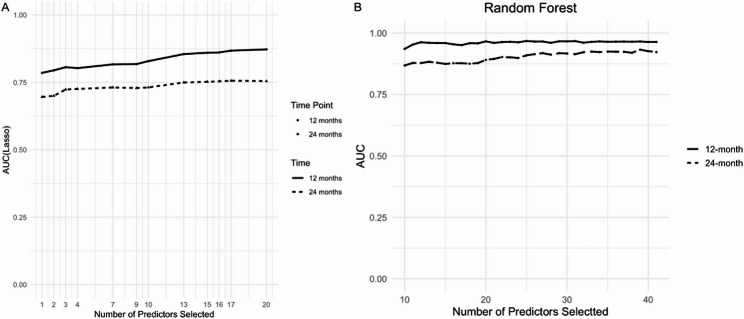
Area under the curve (AUC) according to the number of predictors selected in the Lasso-Cox and random forest models. Solid lines represent 12-month performance and dashed lines represent 24-month performance

Figure 4 presents AUC as a function of the number of predictors in Lasso-Cox (Panel A) and RF (Panel B) models at 12 and 24 months. In Lasso-Cox, the 12-month AUC increases from 0.678 with a single predictor to 0.880 when 20 predictors are included; the 24-month AUC follows a parallel trend, reaching 0.752 (95% CI 0.674–0.830) at 20 predictors. For RF, using all 41 predictors results in a 12-month AUC of 0.990 (95% CI 0.980–1.000) and a 24-month AUC of 0.753 (95% CI 0.674–0.832). Across subsets of 10 to 42 predictors, the 12-month AUC remains above 0.920, while the 24-month AUC stays above 0.750. These combinations indicate that a relatively small set of top-ranked variables can yield optimal discrimination, with marginal gains beyond the top 20 predictors.

Feature importance for RF, GBDT, and SVM models was examined using mean decrease in impurity and SHAP values. In RF models using all predictors (Supplemental Fig. 1) 4D-RVEF had the highest importance, following LAVI, PASP, and TAPSE. When restricted to ReWAS-selected predictors (Supplemental Fig. 2), similar finding was revealed. The non-linear transformer Random Forest (Supplemental Fig. 3) yielded similar top predictors, highlighting the robustness of 4D-RVEF and LAVI. In Supplemental Fig. 4, the importance plot from the RF (non-linear transformer) model with ReWAS confirms that 4D-RVEF and LAVI remain the dominant predictors. In the GBDT model, SHAP summary plots (Supplemental Fig. 5) identified LVEF, RVEDVI, SBP, RV-FWLS, and RVGLS as the most influential features. The ReWAS + GBDT variant (Supplemental Fig. 6) further highlighted RVFWLS, RVIMP, LAVI, PASP, and 4D-RVEF as key contributors. SHAP analysis of the SVM model (Supplemental Fig. 7) showed digoxin usage, MR, TR, LVEF, and beta-blocker use had the largest impact, while the ReWAS + SVM model (Supplemental Fig. 8) emphasized 4D-RVEF, RVFWLS, NYHA class, RVIMP, and RVGLS as dominant predictors.

**Fig. 5:**
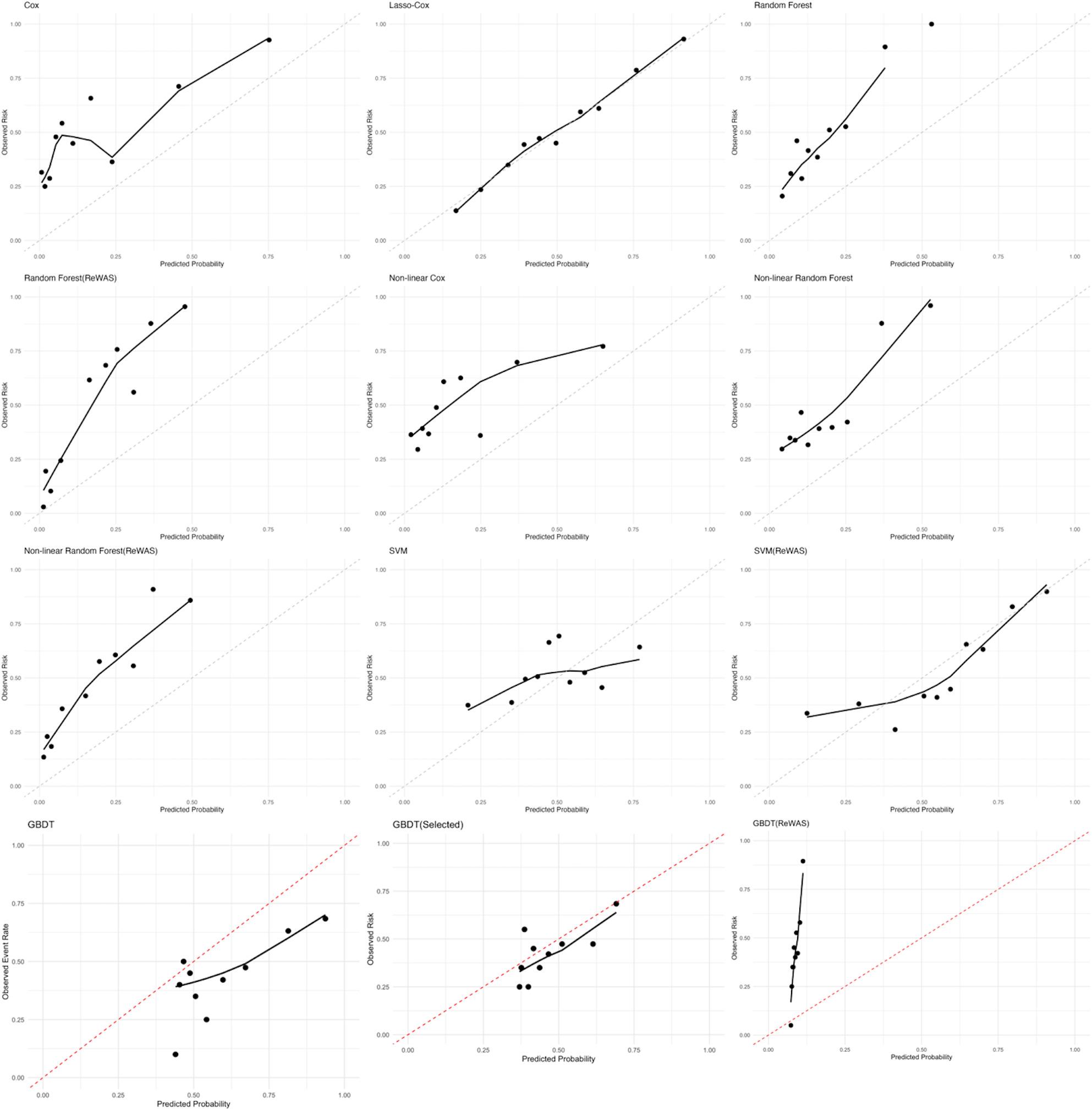
Comparison of calibration curves for predictive models. The x-axis represents predicted risk and the y-axis represents observed event rate. The 45° dashed line indicates perfect agreement between predicted and observed risk. Points closer to the reference line indicate better calibration

Calibration performance was evaluated using calibration plots for all models (Fig. 5). Quantitative calibration measures, including the calibration intercept, calibration slope, and Brier score for each model, are provided in Supplementary Table 8. The Cox and Lasso-Cox models exhibited consistent good calibration. RF and ReWAS + RF models showed underestimated risk across deciles. Non-linear transformer RF with ReWAS selected predictors did not show improved calibration. Both standard and Selected GBDT models revealed restricted calibration (overestimated risk across deciles), whereas the ReWAS + GBDT variant showed underestimated risk across deciles. SVM and ReWAS + SVM models also presented limited calibration. The 45° reference line represents perfect agreement between predicted probability and observed event rate. Deviation above this line indicates systematic overestimation of risk, whereas deviation below the line indicates underestimation. Although several machine-learning models demonstrated high discrimination, the random forest–based models showed clear miscalibration at higher predicted probabilities, suggesting that the predicted absolute risks did not correspond well to observed outcomes. In contrast, the Lasso-Cox model showed closer agreement between predicted and observed risks, indicating more reliable absolute risk estimation.

## Discussion

In this study, we developed and validated multiple prognostic models—including traditional statistical methods and ML algorithms—to predict adverse outcomes in patients with DCM. By integrating 3D echocardiographic parameters with routine clinical data, we aimed to enhance risk stratification in this population. Our findings indicate that while RF models achieved the highest discrimination at 12 months (AUC: 0.990), Lasso-Cox models revealed the best calibration and maintained consistent discrimination over 24 months (AUC: 0.729). Key predictors across models included 4D-RVEF, LAVI, PASP, and TAPSE. In this study, the outcome was defined as a composite clinical deterioration event rather than a single cause-specific endpoint. The components—heart failure rehospitalization, LVAD implantation, and death—represent successive stages along the clinical progression of dilated cardiomyopathy, from decompensation to advanced refractory heart failure and terminal outcome. Accordingly, the models should be interpreted as predicting overall risk of major clinical worsening rather than any individual event. This definition was chosen to capture the trajectory of disease progression and to maintain statistical stability given the limited number of events for each individual component.

Several studies have explored the prognostic value of echocardiographic parameters in DCM. For instance, Toida et al. [30] found that 3D mitral valve tenting geometry assessed via real-time 3D echocardiography (RT3DE) could predict long-term outcomes in DCM patients. Similarly, LAVI has been identified as an independent determinant of left ventricular filling pressures and mitral regurgitation in DCM [31, 32]. However, the integration of advanced 3D echocardiographic measures into comprehensive prognostic models remains limited. Our study addresses this gap by incorporating 3D echocardiographic parameters into both traditional and ML-based prognostic models, thereby enhancing predictive accuracy and clinical applicability.

The incorporation of 3D echocardiographic parameters, particularly 4D-RVEF, into prognostic models offers a more nuanced assessment of right ventricular function, which is crucial in DCM. Unlike 2D measures (which rely on geometric assumptions and single-plane views), 3D echo captures the RV’s complex asymmetrical shape and integrates longitudinal, radial, and anteroposterior components of contraction. This allows direct volumetric measurement of RV ejection fraction without relying on foreshortened views or geometric models [33, 34]. In fact, real-time 3D echocardiography has demonstrated accuracy comparable to cardiac MRI (the gold standard for RV volume) while being faster and more practical at bedside [35].

Considering the calibration performance together with the extremely high AUC value, the RF model may reflect model optimism in a relatively small dataset, leading to unreliable absolute risk estimation. Although the model demonstrated strong discrimination, inaccurate risk probabilities may limit its direct clinical applicability. From a clinical perspective, calibration determines whether the predicted probability can be interpreted as an individual patient’s actual risk. A well-calibrated model allows clinicians to use the estimated risk to guide follow-up intensity, treatment adjustment, or consideration of advanced therapies. In contrast, a poorly calibrated model may systematically overestimate or underestimate risk. Overestimation may lead to unnecessary interventions and overtreatment, whereas underestimation may delay appropriate monitoring or escalation of care. Therefore, despite higher discrimination, models with poor calibration may be less suitable for direct clinical decision-making. Our findings suggest that Lasso-Cox models, balancing discrimination and calibration, may be more suitable for clinical implementation than more complex ML models.The interpretability and parsimony of Lasso-Cox models facilitate their integration into clinical workflows, potentially aiding in personalized risk stratification and management decisions for DCM patients.

The Lasso-Cox model retains a limited set of key predictors, thereby enhancing interpretability and parsimony. These pivotal variables—including 4D-RVEF, LAVI, PASP, and TAPSE —are all readily obtainable in routine practice and mirror pathophysiological processes that critically influence DCM prognosis, such as right-ventricular dysfunction, pulmonary hypertension, and left-atrial remodeling. This not only strengthens the model’s clinical credibility but also provides clinicians with clear focal points for risk assessment, prompting heightened attention to right-heart function and atrial structural changes when managing DCM patients.

Compared with “black-box” algorithms that pursue higher discrimination at the expense of transparency [36], the Lasso-Cox model is better suited for integration into everyday clinical workflows. Because several key predictors identified by the models (e.g., 4D-RVEF, PASP, and TAPSE) reflect right-ventricular dysfunction and hemodynamic deterioration, the prognostic information provided by advanced echocardiographic imaging may be directly actionable in clinical practice. It can be further developed into a practical risk-calculation tool—embedded, for example, within echocardiography reporting systems or electronic health records—to generate real-time, individualized prognostic estimates based on patients’ imaging and clinical data. Such a tool would allow clinicians to identify high-risk DCM patients more accurately, adjust follow-up intervals, optimize therapeutic regimens, or consider early mechanical support or transplantation, thereby improving long-term outcomes. Moreover, our study underscores the importance of calibration and interpretability in prognostic modeling, offering essential guidance for the future development and clinical adoption of similar models. The model is intended to assist clinical decision-making rather than replace physician judgement and should be interpreted in the context of the patient’s overall clinical condition and preferences.

Dilated cardiomyopathy is increasingly recognized as a heterogeneous clinical syndrome rather than a uniform disease entity, with substantial variability in disease progression and outcomes across patients. Recent cohort studies have emphasized the need for improved risk stratification strategies to identify patients at higher risk and to guide management decisions. Our findings are consistent with this evolving perspective. By integrating echocardiographic functional markers with clinical variables, the present models aim to address the prognostic heterogeneity observed in contemporary DCM cohorts and support individualized clinical assessment rather than reliance on single parameters or conventional classifications [37, 38]. Notably, predictive performance declined over time, indicating that a single baseline assessment may be adequate for early risk stratification but less reliable for long-term prognosis. This temporal pattern has practical clinical implications: risk evaluation in DCM should likely be updated periodically during follow-up rather than relying solely on baseline measurements.

Our study employed a comprehensive modeling strategy, comparing 12 prognostic models, including traditional Cox regression, Lasso-Cox, RF, GBDT, and SVM. We utilized internal validation techniques such as bootstrap resampling and OOB estimation, and additionally evaluated model performance at a longer prediction horizon (24 months) within the same cohort. Feature importance was evaluated using SHAP and impurity reduction methods, enhancing the interpretability of our models. Our approach underscores the importance of balancing model complexity with clinical applicability, favoring models that offer both robust predictive performance and ease of interpretation. SHAP importance reflects predictive contribution rather than causal effect; in particular, treatment variables (e.g., digoxin use) likely represent underlying disease severity and confounding by indication rather than therapeutic impact.

A key strength of our study is the integration of advanced 3D echocardiographic parameters with routine clinical data, providing a comprehensive assessment of cardiac function in DCM patients. The comparison of multiple modeling approaches, coupled with rigorous validation techniques, enhances the robustness and generalizability of our findings. However, our study has limitations. The retrospective design and single-center setting may limit the generalizability of our results. In addition, because this was an observational retrospective study, causal relationships between predictors and outcomes cannot be inferred, and the identified associations should be interpreted as prognostic rather than causal. Residual confounding may also be present, as treatment decisions and clinical management during follow-up were not standardized and could not be fully accounted for in the analysis. Additionally, the sample size, while adequate for model development, may not capture the full heterogeneity of the DCM population. Although the number of events was adequate for conventional regression modelling, the overall sample size remains modest for flexible machine-learning algorithms, which typically require larger datasets for stable and transportable prediction structures. Because the composite endpoint combines events of differing severity, the model does not provide cause-specific risk prediction, and outcome-specific or competing-risk analyses will require larger cohorts. The absence of missing predictor data reflects the structured nature of this single-centre specialist registry, where imaging and laboratory assessments were routinely obtained at baseline. In broader real-world or multicentre settings, incomplete data would be expected and would require predefined handling strategies (e.g., standardized acquisition or imputation); therefore, model transportability should be interpreted cautiously. Future studies with larger, multicenter cohorts are warranted to validate our findings and assess their applicability across diverse clinical settings.

Our study highlights the potential of integrating advanced imaging modalities with machine learning techniques to enhance prognostic modeling in DCM. Future research should focus on external validation of our models in larger, multicenter cohorts to confirm their generalizability. Additionally, the development of user-friendly clinical tools incorporating these models could facilitate their adoption in routine practice. Exploring the integration of other imaging modalities, such as cardiac magnetic resonance imaging (MRI), and incorporating genetic and biomarker data may further refine risk stratification in DCM.

## Conclusions

In conclusion, our study demonstrates that integrating 3D echocardiographic parameters with clinical data enhances prognostic modeling in DCM. While RF models achieved the highest discrimination, Lasso-Cox models offered consistently good calibration and interpretability, making them more suitable for clinical application. Our findings support the incorporation of advanced imaging parameters into prognostic models to improve risk stratification and guide management in DCM patients.

## Supplementary Information

Supplementary Material 1.

## Data Availability

The datasets generated and/or analysed during the current study are not publicly available due to the agreement of the hospital that provided the data but are available from the corresponding author on reasonable request.

## Abbreviations

3D: Three-Dimensional
ANOVA: Analysis of Variance
ECG: Electrocardiogram
EHR: Electronic Health Record
FP: Fractional Polynomial
GBDT: Gradient Boosted Decision Tree
LASSO: Least Absolute Shrinkage and Selection Operator
OOB: Out-of-Bag
SHAP: SHapley Additive exPlanations
SVM: Support Vector Machine
