# Supplementary material for: Predicting adverse outcomes in dilated cardiomyopathy using 3D echocardiography: penalised Cox regression versus machine learning
Source: BMC Cardiovasc Disord. 2026 Feb 23;26:264. doi: 10.1186/s12872-026-05645-8 (PMC13037254; doi:10.1186/s12872-026-05645-8)

ONLINE SUPPLEMENTAL FILE

Supplemental Table 1 Optimal parameter for nonlinear variables in polynomial transformations

	variable	p1	p2	AIC	BIC
1	Age	-3	-3	648.066	672.010
2	Body mass index	2	1	641.492	665.436
3	Diastolic blood pressure	-3	-3	645.722	669.666
4	E/e′	3	3	647.704	671.649
5	Heart rate beats	-1	-1	646.850	670.794
6	LAVI	-3	3	641.477	663.027
7	LVDd	3	3	645.033	668.977
8	LVDs	-3	-3	637.176	661.120
9	LVEDVI	1	-3	641.921	665.865
10	LVEF	3	3	641.692	665.636
11	LVESVI	-3	0.5	643.394	667.338
12	PAPS	-3	3	646.467	668.017
13	RVFAC	-3	-3	640.501	664.445
14	RVIMP	-3	-3	645.332	666.882
15	RVDd-base	0.5	0.5	646.368	670.312
16	RVDd-mid	3	-3	638.236	662.181
17	RVDd	3	3	648.854	672.798
18	RVESVI	-3	3	640.520	664.464
19	RVFWLS	3	3	641.349	662.899
20	RVGLS	3	-3	644.539	666.089
21	RVLd	-3	-3	647.797	671.742
22	RVSVI	2	-1	639.596	663.541
23	SBP	-3	-3	643.865	667.809
24	TAPSE	-3	-3	643.857	665.407
25	TV-S’	-3	-3	642.056	666.000
26	1_RVESVI	3	-3	640.134	664.078
27	4D_RVEF	-3	3	645.894	667.444
28	4D_RVFAC	3	3	647.850	671.795
29	4D_TAPSE	-3	-3	642.406	666.351

Where p1 denotes the first term of the polynomial and p2 denotes the second term. If the expressions for p1 and p2 are identical, then p2 is further multiplied by log(x). AIC stands for the Akaike Information Criterion, and BIC stands for the Bayesian Information Criterion.

Supplemental Table 2 Results of Cox regression with backward selected

Variable	Coefficient	HR (95% CI)	P-value
BMI	-0.043	0.958 (0.915, 1.003)	0.069
SBP	0.021	1.022 (1.004, 1.039)	0.013
DBP	-0.032	0.968 (0.947, 0.990)	0.0046
HR	0.014	1.014 (1.000, 1.028)	0.046
NYHA Class	0.537	1.711 (0.972, 3.012)	0.063
ACEI ARB	0.72	2.054 (1.094, 3.855)	0.025
Loop Diuretics	-0.667	0.513 (0.256, 1.031)	0.061
Digoxin	-0.412	0.662 (0.437, 1.004)	0.053
MR	0.519	1.680 (1.088, 2.596)	0.019
RVDd_mid	-0.028	0.972 (0.942, 1.003)	0.071
RV_GLS	-0.086	0.917 (0.858, 0.981)	0.011
RV_FWLS	0.064	1.066 (1.010, 1.125)	0.021
4D_RVEF	-0.101	0.904 (0.876, 0.933)	2.90×10^-10
RVEDVI	0.008	1.008 (1.002, 1.014)	0.015
RVESVI	0.104	1.110 (1.001, 1.231)	0.048
1_RVESVI	0.12	1.127 (1.015, 1.251)	0.025

Supplemental Table 3 Lasso-Cox model selected variables and coefficients

Variable	Coefficient
Age	0.000046
BMI	-0.008
ACEI_ARB	0.103
Digoxin	-0.045
LVDs	-0.00019
LVEF	-0.004
MR	0.093
RV_GLS	-0.001
4D_RVEF	-0.055
1_RVESVI	0.002

Supplemental Table 4 RSF model parameters and performance comparison

Item	All variables RSF	ReWAS-selected variables RSF	Non-linear transformed variables RSF
Sample size	196	196	196
Number of deaths	37	37	37
Number of trees	500	500	1000
Terminal node size	15	15	15
Average number of terminal nodes	7.97	7.82	7.709
Variables tried at each split	7	3	8
Total number of variables	41	9	61
Resampling method	swor	swor	swor
Resample size	124	124	124
Analysis	RSF	RSF	RSF
Family	Survival	Survival	Survival
Splitting rule	logrank	logrank	logrank
Number of random split points	10	10	10
OOB CRPS	0.8385	0.8278	0.7843
OOB standardized CRPS	0.0699	0.069	0.0654
OOB performance error	0.2745	0.2621	0.2029

RSF stands for Random Survival Forest, and “swor” indicates sampling without replacement. Family = surv denotes that the model is fit for time-to-event (survival) data, while the “logrank random” splitting rule uses randomly chosen split points evaluated by the log-rank test statistic. OOB refers to out-of-bag estimation, CRPS is the continuous ranked probability score (with lower values indicating better calibration and discrimination), and stand. CRPS is the CRPS value standardized to facilitate comparison across different scales.

Supplemental Table 5 Non-linear transformed cox model coefficients and hazard ratios

Variable	Coefficient	HR((95%)	P-value
ACEI ARB	0.615	1.800 (1.055, 3.240)	0.032
Loop Diuretics	-0.559	0.568 (0.318, 1.083)	0.092
MR	0.465	1.371 (0.970, 2.278)	0.011
SBP	2.021	7.545 (0.893, 63.775)	0.063
DBP	-2.358	0.113 (0.021, 0.620)	0.012
HR	0.906	2.474 (0.811, 7.509)	0.11
4D_RVEF	-4.039	0.040 (0.012, 0.130)	2×10^-10
LVDs_fpn3_0	3.375	29.230 (0.950, 899.620)	0.053
LVDs_fpn3_0_log	2.424	11.290 (0.750, 169.400)	0.079
TAPSE_fpn2_0	-2.935	0.053 (0.003, 0.946)	0.046
TAPSE_fpn2_0_log	-2.129	0.119 (0.017, 0.847)	0.034
TV_S_fpn3_0	43.139	5.024×10^15 (3.037×10^2, 8.309×10^28)	0.006
TV_S_fpn3_0_log	43.521	7.07415 (2.507×10^2, 1.996×10^29)	0.008
RV_GLS_fp3_0	1.886	6.596 (2.990, 14.565)	3.1×10^-6
RV_GLS_fp3_0_log	2.627	13.833 (4.490, 42.665)	4.8×10^-6
RV_FWLS_fp3_0	-2.002	0.140 (0.020, 0.875)	0.036
RV_FWLS_fp3_0_log	-2.701	0.067 (0.009, 0.519)	0.01
RVSVI_fp2_0	0.622	1.862 (1.262, 2.747)	0.002
RVSVI_fpn1_0	1.091	2.977 (1.622, 5.461)	0.00043

Fractional-polynomial (FP) transformations are encoded in variable names by the pattern _fpP1_P2 (and _fpP1_P2_log when P_1 = P_2). Different numeric values therefore capture different polynomial powers of the standardized variable in the nonlinear Cox model.

Supplemental Table 6 GBDT survival model parameters

Parameter	GBDT (all selected features)	ReWAS + GBDT
Objective function	survival:cox	survival:cox
Learning rate (eta)	0.05	0.05
Maximum tree depth	6	6
Subsample ratio	0.7	0.7
Column subsample (by tree)	0.8	0.8
Parameter validation	TRUE	TRUE
Number of boosting rounds	500	800
Number of features used	10	8

survival:cox, Cox proportional hazards objective function; subsample, fraction of observations randomly sampled to grow each tree; column subsample (by tree), fraction of predictors randomly sampled at each tree.

Supplemental Table 7 Support Vector Machine (SVM) model parameters

Parameter	SVM (selected features)	ReWAS + SVM
Survival SVM approach	Regression	Regression
Kernel type	Additive kernel (add_kernel)	Additive kernel (add_kernel)
Optimization solver	quadprog	quadprog
Number of support vectors retained	43	92
gamma.mu parameter value	0.1	0.1
Response variable	Surv(ST, event)	Surv(ST, event)
R package version	survivalsvm 0.0.5	survivalsvm 0.0.5

Surv(ST, event), survival outcome defined by follow-up time (ST) and event indicator; add_kernel, additive kernel function; quadprog, quadratic programming optimization solver; gamma.mu, regularization parameter controlling the margin penalty; support vectors, observations defining the decision boundary in the SVM model.

Supplemental Table 8 Brier score and calibration intercept/slope for prediction models

Model	Brier score (95% CI)	intercept	slope
Cox	0.319 (0.256, 0.382)	0.35	0.773
Lasso-Cox	0.205 (0.174, 0.235)	-0.015	1.037
Random Forest	0.320 (0.265, 0.375)	0.247	1.298
Random Forest (ReWAS)	0.308 (0.258, 0.358)	0.141	1.888
Non-linear Cox	0.346 (0.287, 0.405)	0.367	0.070
Non-linear Random Forest	0.321 (0.265, 0.376)	0.146	0.724
Non-linear Random Forest (ReWAS)	0.272 (0.229, 0.315)	0.178	0.826
SVM	0.291 (0.265, 0.316)	0.336	0.378
SVM (ReWAS)	0.365 (0.327, 0.402)	0.260	0.587
GBDT	0.220 (0.203, 0.237)	0.058	0.687
GBDT (selected)	0.269 (0.247, 0.292)	-0.047	1.037
GBDT (ReWAS)	0.549 (0.465, 0.633)	-0.347	2.273

Supplemental Figure 1 Feature importance plot from random survival forest using ReWAS selected variables

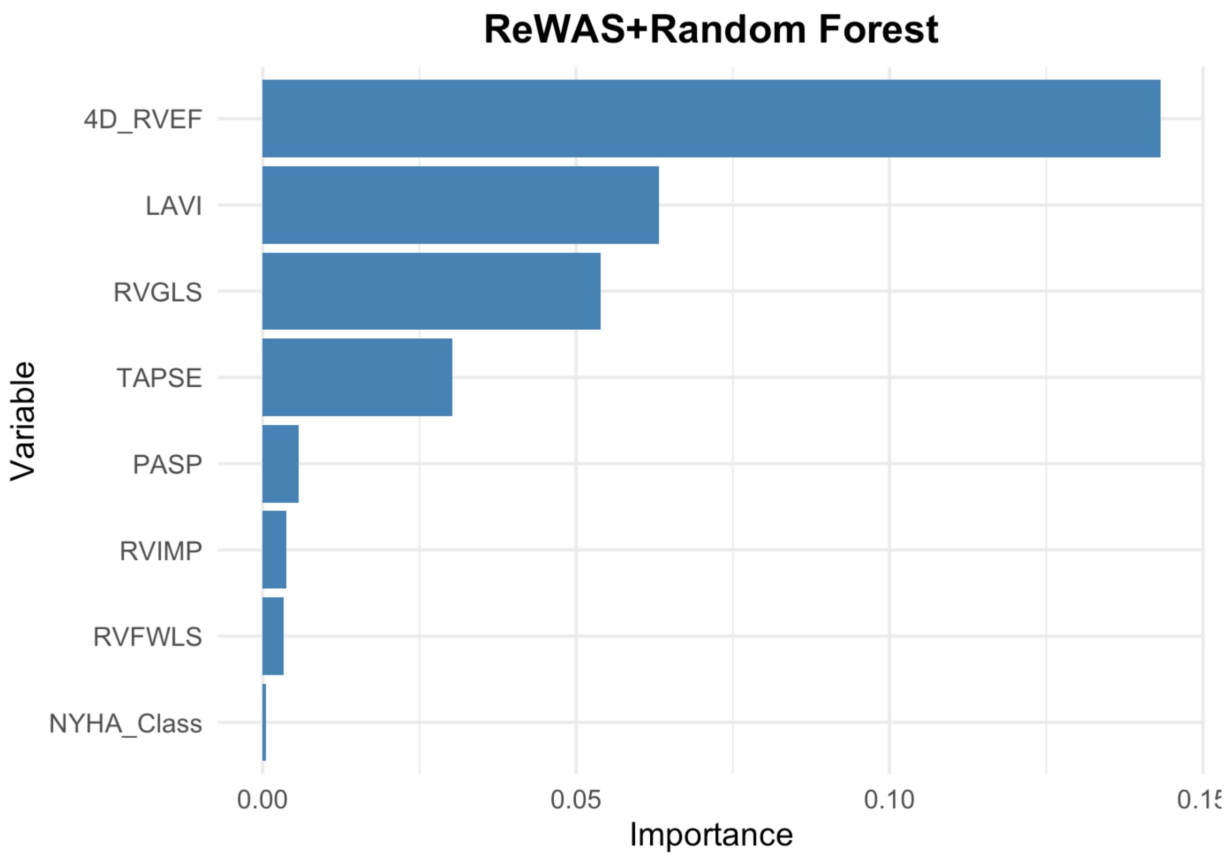

Supplemental Figure 2 Feature importance plot from random survival forest of all variables

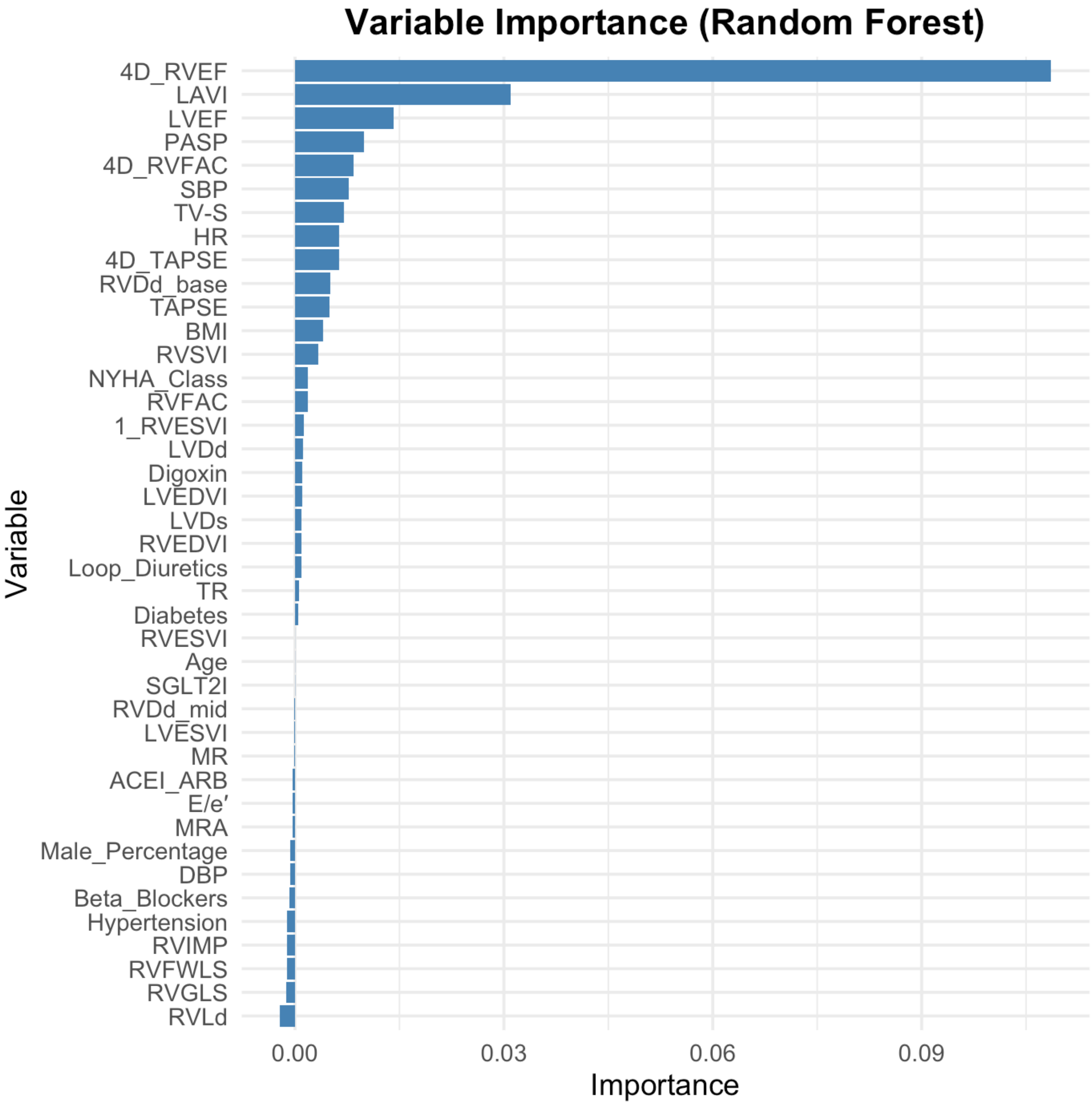

Supplemental Figure 3 Feature importance plot from random survival forest using all non-linearly transformed variables

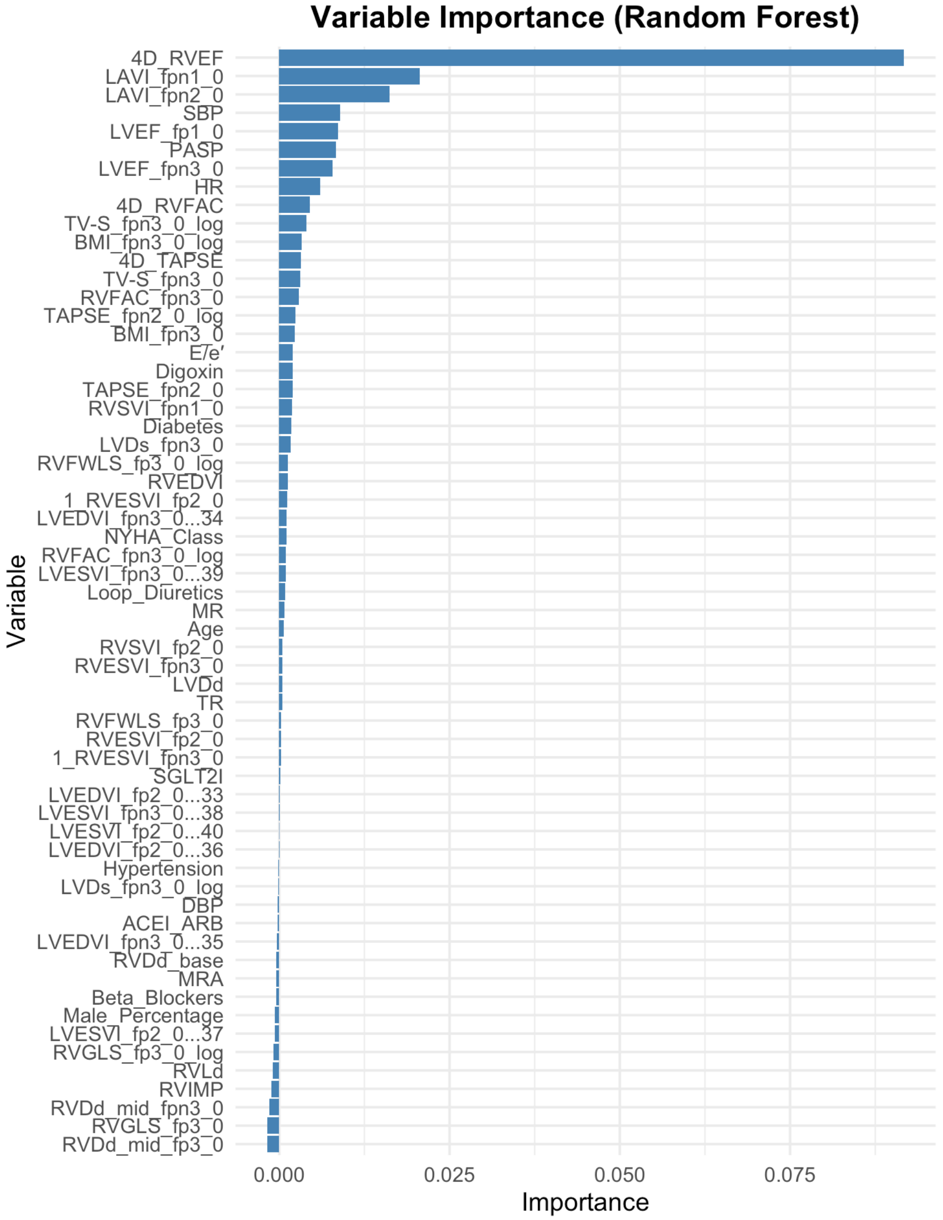

Supplemental Figure 4 Feature importance plot from random survival forest using fold change selected variables with non-linear transformation

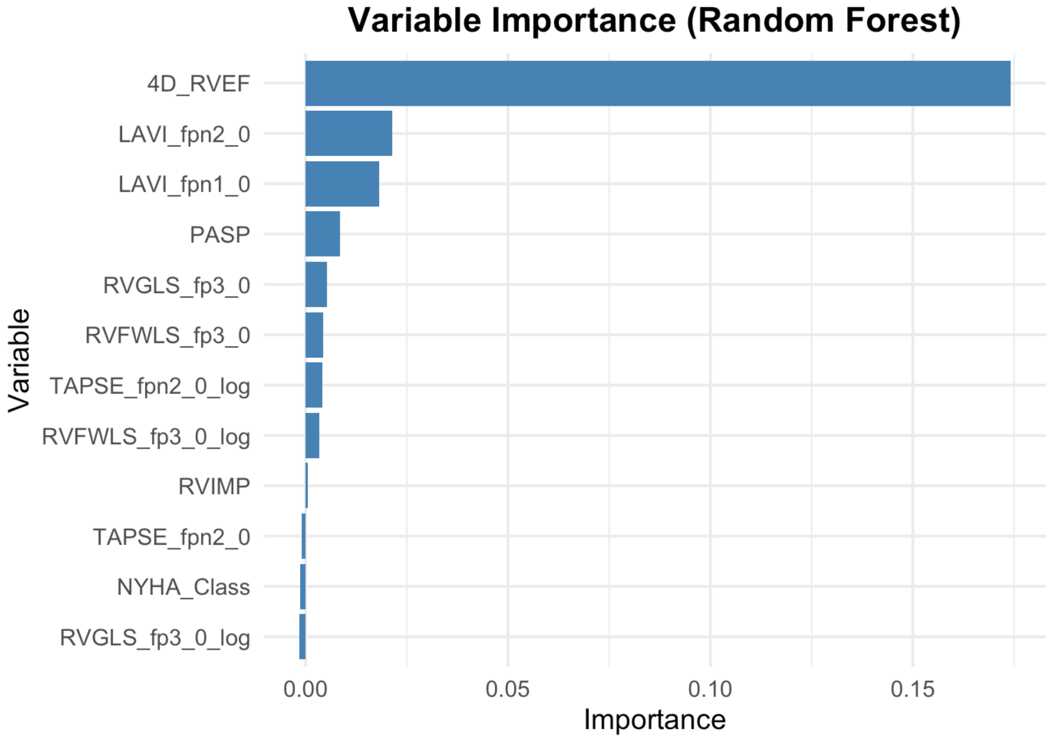

Supplemental Figure 5 SHAP summary plot showing feature importance and impact on GBDT model predictions. *The x-axis shows the SHAP value (contribution of a variable to the predicted risk), and the y-axis lists predictors ranked by importance. Color indicates the value of the variable (red = higher value, blue = lower value). The bar chart on the right shows the mean absolute SHAP value for each predictor.*

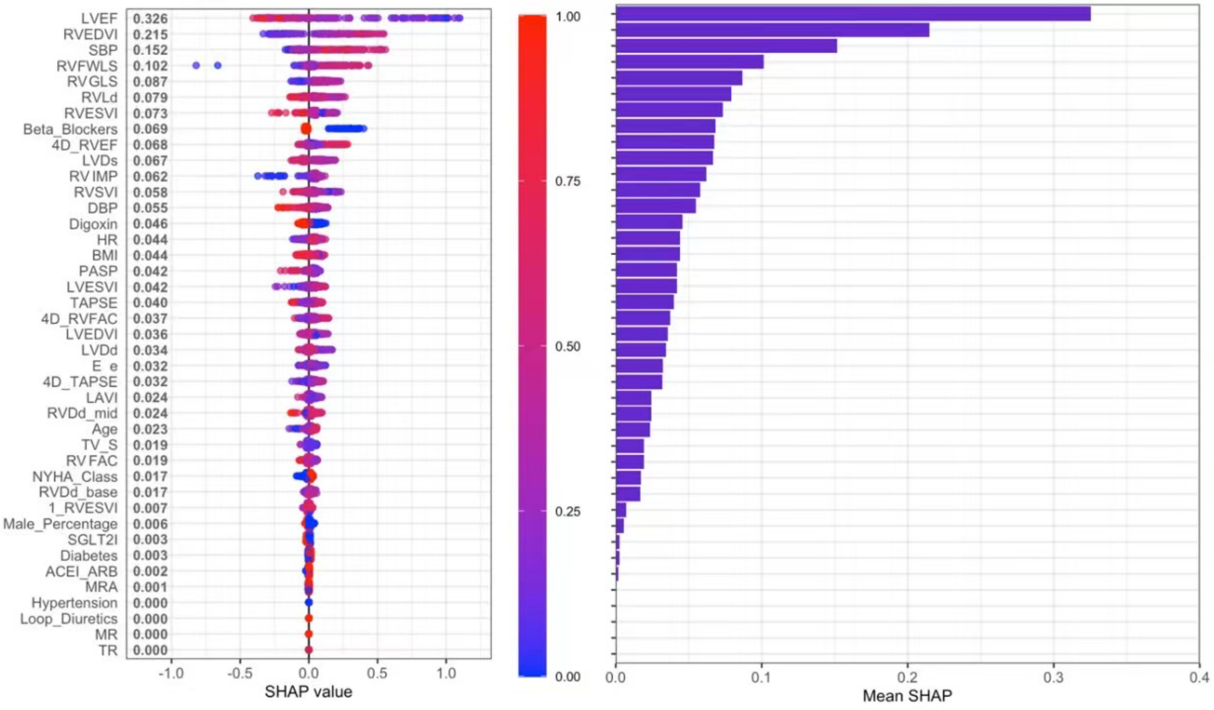

Supplemental Figure 6 SHAP Summary Plot Showing Feature Importance and Impact on ReWAS+GBDT model predictions.

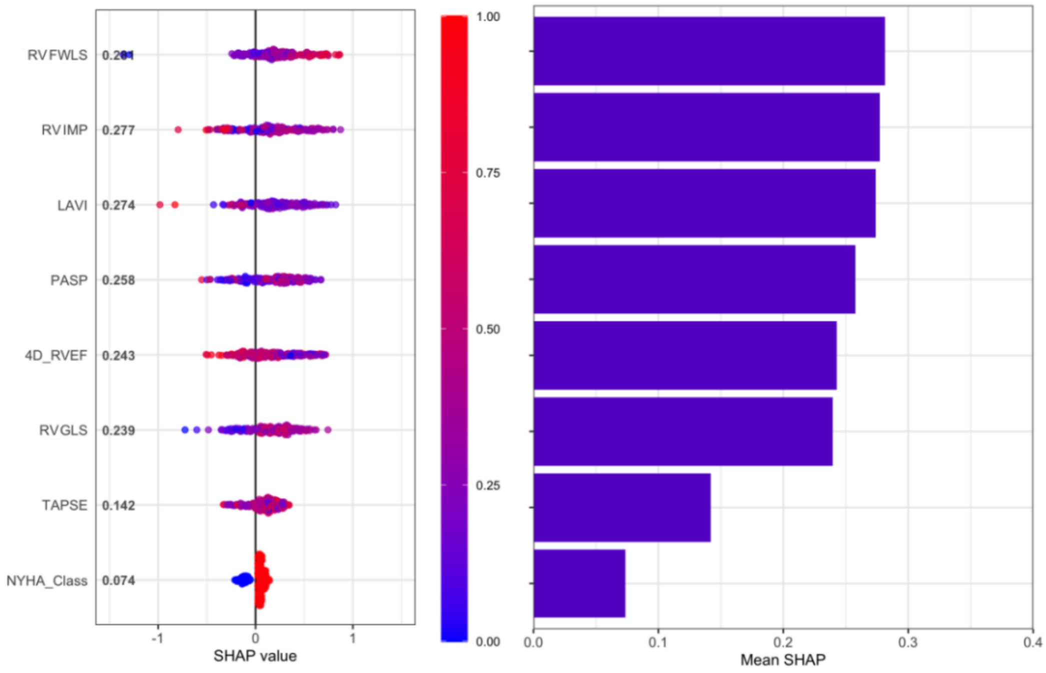

Supplemental Figure 7 SHAP summary plot showing feature importance and impact on SVM model predictions. *Each point represents one patient. The x-axis shows the SHAP value (contribution of a variable to the predicted risk), and the y-axis lists predictors ranked by importance. Color indicates the value of the variable (yellow = higher value, dark = lower value). The bar chart on the right shows the mean absolute SHAP value for each predictor.*

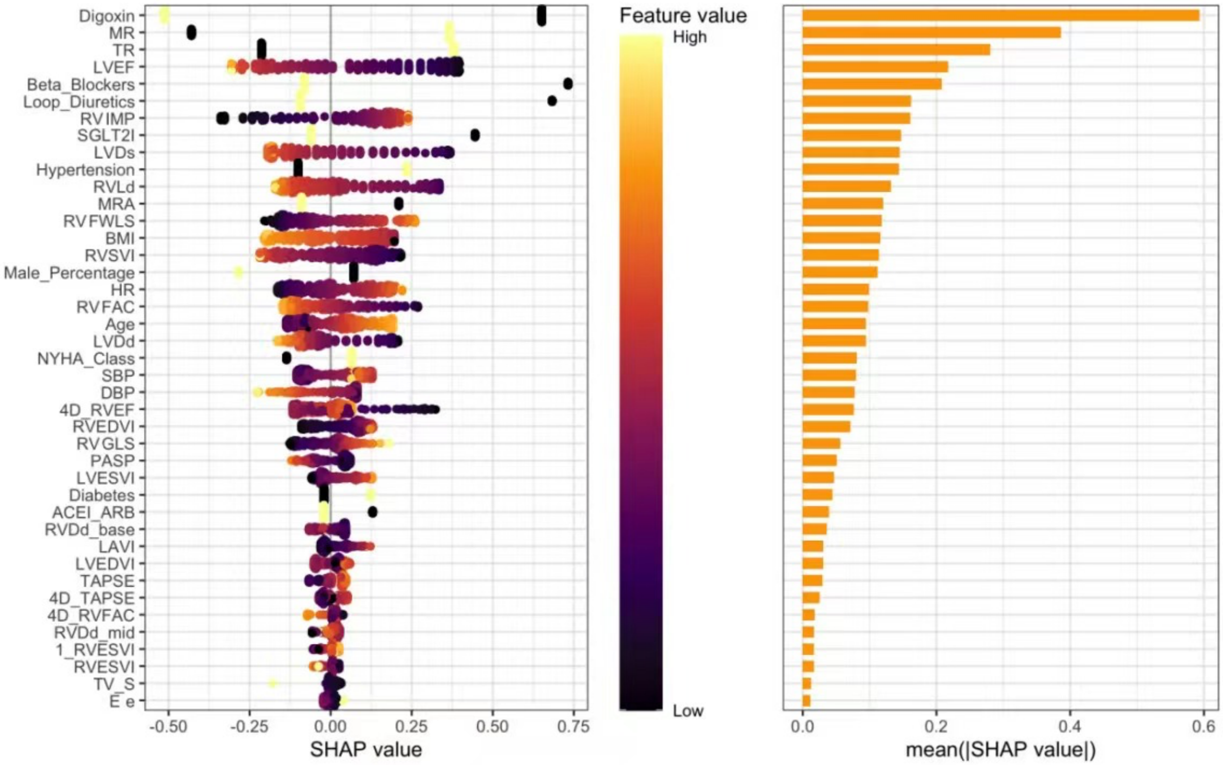

Supplemental Figure 8 SHAP Summary Plot Showing Feature Importance and Impact on ReWAS+SVM model predictions.

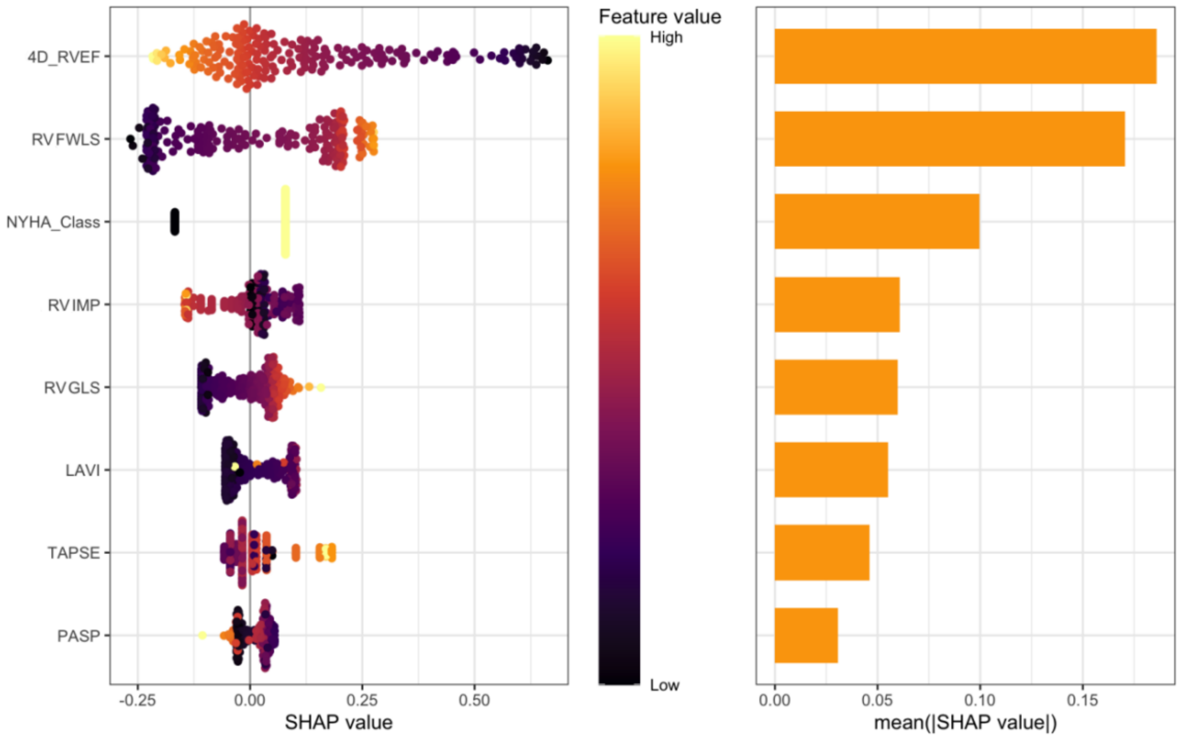

Supplement: Supplementary file 1 — Supplementary Material 1. [file 12872_2026_5645_MOESM1_ESM.pdf]
